# Un enemigo oculto: tratando de entender el síndrome hemofagocítico en niños menores de 5 años en una institución de alta complejidad del suroccidente de Colombia

**DOI:** 10.7705/biomedica.7526

**Published:** 2024-12-23

**Authors:** Sofía Martínez, Jacobo Triviño, Oriana Arias, Diego Medina, Alexis Franco, Jaime Patiño, Paola Pérez, Harry Pachajoa, Pamela Rodríguez, Manuela Olaya-Hernández

**Affiliations:** 1 Centro de Investigaciones Clínicas, Fundación Valle del Lili, Cali, Colombia Fundación Valle del Lili Fundación Valle del Lili Cali Colombia; 2 Hemato-Oncología Pediátrica, Departamento de Pediatría, Fundación Valle del Lili, Cali, Colombia Fundación Valle del Lili Hemato-Oncología Pediátrica Departamento de Pediatría Fundación Valle del Lili Cali Colombia; 3 Departamento de Infectología Pediátrica, Fundación Valle del Lili, Cali, Colombia Fundación Valle del Lili Departamento de Infectología Pediátrica Fundación Valle del Lili Cali Colombia; 4 Facultad de Ciencias de la Salud, Universidad ICESI, Cali, Colombia Universidad ICESI Facultad de Ciencias de la Salud Universidad ICESI Cali Colombia; 5 Departamento de Pediatría, Alergología e Inmunología Pediátrica, Fundación Valle del Lili, Cali, Colombia Fundación Valle del Lili Departamento de Pediatría Alergología e Inmunología Pediátrica Fundación Valle del Lili Cali Colombia

**Keywords:** síndrome hemofagocítico, pediatría, informes de casos., Lymphohistiocytosis, hemophagocytic, pediatrics, case reports.

## Abstract

**Introducción.:**

El síndrome hemofagocítico es una condición poco reconocida, pero con gran mortalidad en la población pediátrica. Se caracteriza por la activación excesiva de células inmunitarias y la liberación de citocinas, lo cual causa inflamación persistente. Puede ser primario o secundario, asociado con diversos factores.

**Objetivo.:**

Describir 12 casos clínicos de niños menores de cinco años con síndrome hemofagocítico en una institución de alta complejidad del suroccidente de Colombia.

**Materiales y métodos.:**

Se presenta una serie retrospectiva de 12 casos de síndrome hemofagocítico en menores de cinco años en una institución de alta complejidad de Colombia entre el 2019 y el 2022.

**Resultados.:**

La mediana de la edad de los pacientes fue de un año, 7 eran de sexo masculino. La fiebre y la esplenomegalia fueron las manifestaciones clínicas más comunes en 11 pacientes. Los hallazgos de laboratorio predominantes fueron: hiperferritinemia en 11 casos, hipertrigliceridemia en 10, bicitopenia en 6 y pancitopenia en 2. Se observó aumento de la lactato-deshidrogenasa en 11 casos. Siete pacientes se sometieron a estudios genéticos. En cuanto al tratamiento, el protocolo HLH-2004 completo se cumplió en 5 pacientes. Tres casos reportados en este trabajo recibieron trasplante de progenitores hematopoyéticos. Tres pacientes fallecieron.

**Conclusión.:**

Se resalta la complejidad del síndrome hemofagocítico y, más aún, en niños menores de cinco años, ya que la baja prevalencia de la enfermedad y su presentación clínica inespecífica contribuyen al subdiagnóstico. Se destaca la importancia de considerar los factores desencadenantes, la evaluación genética para un diagnóstico preciso y temprano, la necesidad de un enfoque multidisciplinario y la consideración oportuna del trasplante de progenitores hematopoyéticos para mejorar la morbimortalidad.

## Abstract (en)

**Introduction.:**

Hemophagocytic syndrome is an under-recognized condition with high mortality in the pediatric population. It is characterized by excessive activation of immune cells and cytokine release, leading to persistent inflammation. Hemophagocytic syndrome can be primary or secondary and associated with different triggers.

**Objective.:**

To describe 12 clinical cases of children under five years of age with hemophagocytic syndrome in a high-complexity institution in southwestern Colombia.

**Materials and methods.:**

We present a retrospective series of 12 cases of hemophagocytic syndrome in children under five years of age treated at a high-complexity institution in Colombia between 2019 and 2022.

**Results.:**

The median age of the patients was one year and 7 were male. Fever and splenomegaly were the most common clinical manifestations observed in 11 of the patients. The predominant laboratory findings included hyperferritinemia (n = 11), hypertriglyceridemia (n = 10), bicytopenia (n = 6), and pancytopenia (n = 2). Eleven cases had elevated lactate dehydrogenase levels. Genetic studies were conducted in 7 patients. Regarding treatment, the full HLH-2004 protocol was administered to 5 cases, while 3 underwent hematopoietic stem cell transplantation. Three patients died.

**Conclusion.:**

We highlight the complexity of the hemophagocytic syndrome, especially in children under five years old, because the low prevalence and non-specific clinical presentation of the disease contribute to its underdiagnosis. Emphasis is placed on identifying triggers, performing genetic evaluation for accurate and early diagnosis, adopting a multidisciplinary approach, and considering early hematopoietic stem cell transplantation to improve morbidity and mortality outcomes.

El síndrome hemofagocítico fue descrito entre 1935 y 1939 por Scott y Robb-Smith ^1^^,^^2^. A lo largo de los años, mantiene gran relevancia en el ámbito clínico por la mortalidad que ocasiona en la población pediátrica, a pesar de ser una enfermedad poco reconocida y subdiagnosticada ^1^^,^^3^.

En la fisiopatología del síndrome hemofagocítico se ha descrito la activación excesiva de linfocitos T CD8+, células asesinas naturales (*natural killer*, NK) y macrófagos, la cual induce la liberación de citocinas y conlleva inflamación persistente ^1^. La enfermedad puede ser primaria (familiar) o secundaria (adquirida). Teniendo en cuenta que existe una forma de síndrome hemofagocítico primario sin un defecto genético identificado, en este estudio nos referimos al síndrome hemofagocítico primario clásico.

El síndrome hemofagocítico primario está asociado con variantes patógenas en genes específicos, como *PRF*_*1*_ (FHLH-2), *UNC13D* (FHLH- 3), *STX*_*11*_ (FHLH-4) y *STXBP2* (FHLH-5) ^1^^-^^3^. La forma secundaria de la enfermedad se ha asociado con otros errores innatos de la inmunidad, enfermedades reumatoideas, infecciones virales [principalmente por el virus de Epstein-Barr (EBV) ^3^, y otros menos frecuentes como CMV, HBV, VZV, HHV y el de la rubéola] y fúngicas, enfermedades invasivas y neoplasias (con predominio de los linfomas).

La prevalencia y la incidencia varían según la región geográfica. La incidencia en niños es baja (1,2 casos por millón de habitantes por año), ^1^ pero con tasas de mortalidad del 8 hasta el 22 % ^3^, especialmente en poblaciones con algún grado de consanguinidad ^3^.

En la actualidad, se utilizan las guías para el diagnóstico y el tratamiento de la enfermedad de la *Histiocyte Society, hemophagocytic lymphohistiocytosis*-2004 ^1^^,^^2^. El tratamiento se ha condicionado a la causa etiológica o a los factores desencadenantes subyacentes y a la gravedad de la enfermedad ^3^^,^^4^. Se plantea la relevancia de un tratamiento temprano y continuo para reducir la mortalidad ^4^.

El objetivo de este estudio fue describir 12 casos clínicos de niños menores de cinco años con síndrome hemofagocítico en una institución de alta complejidad del suroccidente de Colombia.

## Materiales y métodos

En este estudio retrospectivo, se describen los casos clínicos de 12 pacientes menores de cinco años con diagnóstico confirmado de síndrome hemofagocítico, los cuales cumplieron, al menos, con 5 de los 8 criterios de la HLH-2004. Los casos fueron seleccionados de los registros médicos de la Fundación Valle del Lili en Cali, Colombia, durante el periodo de cuatro años (enero del 2019 a diciembre del 2022).

Se revisaron los registros médicos electrónicos de cada paciente para recopilar los datos demográficos, las características clínicas y paraclínicas, el tratamiento recibido y el resultado del seguimiento. Estos datos se analizaron de forma descriptiva; se presentan las características más relevantes de los pacientes, y se resumen los hallazgos clínicos y de laboratorio.

### Consideraciones éticas

Este estudio se presentó con el número 2023.217 y fue aprobado por el Comité de Ética en Investigación Biomédica de la Fundación Valle del Lili. Los principios éticos de la Declaración de Helsinki se respetaron en todas las etapas del estudio. Según las directrices del Comité de Ética en Investigación Biomédica y la clasificación del estudio como “sin riesgo”, no se solicitó el consentimiento informado.

## Resultados

Durante los últimos años, se ha incrementado la identificación temprana del síndrome hemofagocítico, lo cual ha permitido la instauración de un tratamiento oportuno según su causa. Sin embargo, los niños menores de cinco años son un reto, no solo diagnóstico, sino etiológico. Se revisaron las historias clínicas de todos los pacientes pediátricos atendidos en la Fundación Vale del Lili entre los años 2019 y 2022 y se identificaron los menores de cinco años para este análisis. De los 12 pacientes incluidos, 6 tenían entre 1 y 2 años, y 7 eran de sexo masculino.

De los 12 pacientes analizados, 11 presentaron un factor desencadenante infeccioso que resultó en el desarrollo del síndrome hemofagocítico y cumplieron con, al menos, cinco de los ocho criterios diagnósticos del protocolo HLH-2004. Once de los pacientes presentaron fiebre, con una duración mediana de 7 días. La mediana del tiempo de hospitalización fue de 18 días.

En 11 pacientes, se analizaron las inmunoglobulinas séricas; solo uno (caso 1) presentó hipogammaglobulinemia de IgG, mientras que los demás tuvieron niveles normales de IgA, IgG e IgM.

El paciente número 12 no presentaba manifestaciones clínicas de síndrome hemofagocítico y fue hospitalizado debido a un diagnóstico genético de síndrome linfoproliferativo ligado al cromosoma X, con antecedentes de mortalidad de sus hermanos varones. A los 11 pacientes restantes, se les solicitó solamente la secuenciación de su exorna. Sin embargo, por dificultades administrativas, solo se pudo realizar en 7 de los 11 pacientes. De estos, 5 presentaron variantes patógenas: cuatro, asociadas con síndrome hemofagocítico primario u otros errores innatos de la inmunidad con manifestación de síndrome hemofagocítico, y una (caso 5) relacionada con otro tipo de error innato de la inmunidad. Las variantes específicas se describen en cada caso. El estudio del exorna de los casos 1 y 8 no mostró variantes patógenas.

Se practicó aspirado de médula ósea a todos los pacientes, para determinar la presencia de macrófagos activados. De los 12 pacientes, 5 tenían macrófagos activados en la médula ósea, mientras que en 7 no se detectaron.

En el presente estudio, se realizaron los estudios de laboratorio necesarios para evaluar los criterios diagnósticos de síndrome hemofagocítico en todos los pacientes participantes. Se obtuvieron los siguientes resultados: la mediana de leucocitos fue de 8.200 células/μl (IQR = 6.710 - 16.943); la de neutrófilos, 2.045 células/μl (IQR = 1.137,5 - 3.032,5), y la de plaquetas, de 188.500 células/μl (IQR = 80.500 - 306.750).

La mediana de la hemoglobina fue de 9,6 g/dl (IQR = 8,98 - 10,80), la del fibrinógeno, 209 mg/dl (IQR = 111 - 292), la de los triglicéridos, 382 mg/dl (IQR = 371 - 391), y la de la ferritina, 3.623 ng/ml (IQR = 1.716 - 6.265). La lactato-deshidrogenasa tuvo una media de 789 U/L (IQR = 622 - 1.289). En el resto de las mediciones las medianas fueron de 789 U/L (IQR = 622 - 1.289). Solo en cuatro pacientes se estudió la citotoxicidad de las células NK, por las dificultades logísticas del procesamiento en nuestro medio, dos de los cuales la tenían alterada: uno de ellos con síndrome hemofagocítico primario y el otro sin estudio genético.

Durante el periodo de estudio, se evaluó la posible infección por SARS- CoV-2 en el contexto de la pandemia del 2020. A todos los pacientes se les practicaron pruebas moleculares (PCR) rutinarias para la detección del virus. De los 12 pacientes evaluados, solo 2 resultaron positivos para SARS-CoV-2. Por otra parte, 5 pacientes presentaron infecciones por citomegalovirus y otros 5 por el virus de Epstein-Barr. Un único paciente (caso 9) presentó infección simultánea por ambos virus. Solo en dos pacientes se evidenció infección por dengue.

Finalmente, a todos los pacientes se les diagnosticó síndrome hemofagocítico. El tratamiento se inició con inmunoglobulina humana inespecífica intravenosa o con dexametasona. En los casos en los cuales persistió la actividad desreguladora propia de la enfermedad, en las primeras 72 horas después del diagnóstico se decidió cumplir el protocolo HLH-2004, según las directrices institucionales. El protocolo HLH-2004 completo se utilizó en 5 pacientes. Además, 9 recibieron inmunoglobulina intravenosa a una dosis inmunomoduladora de 1 mg/kg y 11 fueron tratados con dexametasona. Asimismo, 3 pacientes fueron sometidos a trasplante de progenitores hematopoyéticos obtenidos de donantes haploidénticos. Lamentablemente, 3 de los 12 pacientes fallecieron durante el periodo del estudio.

## Caso 1

Se trata de una niña de dos años, sin antecedentes clínicos relevantes y con vacunación completa, que presentó un cuadro clínico de cinco días de fiebre sin un foco aparente y hepatoesplenomegalia, por lo cual se le practicaron exámenes ampliados de laboratorio.

Los resultados evidenciaron leucopenia (2.000 células/μl), neutropenia (400.000 células/μl) y trombocitopenia (61.000 células/μl). El valor de la hemoglobina fue de 10 g/dl, el fibrinógeno estaba disminuido (54 mg/dl). Además, presentaba hipertrigliceridemia (393 mg/dl), y aumento de la lactato- deshidrogenasa (3.666 Ul/L), de la ferritina (*33.328* ng/ml) y de las enzimas hepáticas.

La paciente presentaba anticuerpos IgG e IgM contra el virus de Epstein- Barr, lo que confirmó una infección activa con carga viral muy elevada (más de un millón de copias por mililitro). Los estudios de inmunidad humoral y celular estaban en los rangos normales para la edad.

La paciente cumplía con los criterios para el diagnóstico de síndrome hemofagocítico por lo que se le inició tratamiento con 1 mg/kg de inmunoglobulina humana inespecífica intravenosa, sin mejoría clínica. Se trató según el protocolo HLH-2004, el cual completó sin complicaciones.

Se hizo un estudio genético con secuenciación de su exorna, sin evidencia de variantes patógenas; no se practicaron estudios genéticos por exorna o genoma a sus padres y abuelos. La paciente presentó una buena evolución clínica, sin recaídas después de finalizar el protocolo. No requirió trasplante de progenitores hematopoyéticos.

## Caso 2

Se trata de una niña de un año, con antecedentes de sepsis neonatal temprana y hospitalización por encefalitis aguda diseminada asociada con el SARS-CoV-2 con secuelas neurológicas (ataxia troncular).

Presentaba múltiples lesiones diseminadas de molusco contagioso y evidencia de linfopenia con predominio de CD4, y aumento de linfocitos B y células NK. Las inmunoglobulinas séricas estaban en el rango de los valores normales. Estaba en tratamiento profiláctico con trimetoprim-sulfametoxazol.

Fue hospitalizada por un cuadro clínico consistente en síndrome febril, asociado con irritabilidad y tos seca persistente. En el examen físico, se detectó hepatoesplenomegalia. Los resultados de los exámenes de laboratorio demostraron: leucocitosis (17.880 células/μl), anemia (hemoglobina, 10 g/dl), hipertrigliceridemia (738 mg/dl), hiperferritinemia (*738* ng/ml) y alteración de la citotoxicidad.

La paciente cumplía con los criterios diagnósticos del síndrome hemofagocítico, por lo cual se inició inmunoglobulina humana inespecífica intravenosa. Por persistencia del cuadro clínico, se instauró el protocolo HLH-2004 completo. Se hizo el estudio genético de su exorna, que evidenció variantes heterocigotas, probablemente patógenas, en el gen *PRF1* en el loci 10q22.1 (NM_001083116.3): c.160C>T (p.Arg54Cys) y c.350_356delTGGCCCGinsATGC (p.Val117_Arg119delinsAspAla).

Al intentar disminuir la dosis de quimioterapia, la paciente recayó, por lo cual se sometió a trasplante de progenitores hematopoyéticos y su evolución fue adecuada. No se ha logrado hacer estudios genéticos por exorna o genoma a sus padres y abuelos.

## Caso 3

Se trata de una niña de diez meses de vida, remitida por un cuadro clínico de un mes y medio de evolución de fiebre intermitente asociada con síntomas de las vías respiratorias altas.

Fue hospitalizada en otra institución por presentar fiebre persistente asociada con síntomas respiratorios y hepatoesplenomegalia; se detectó infección por virus de la parainfluenza de tipo 3 y coronavirus.

Inicialmente, se consideró que estaba cursando con un síndrome de Kawasaki atípico, por lo cual el Servicio de Reumatologia prescribió esteroides e inmunoglobulina humana inespecífica intravenosa. Al no presentar una evolución satisfactoria, la paciente fue remitida a la Fundación Valle del Lili, donde se confirmó la hepatoesplenomegalia.

Otros hallazgos incluyeron el aumento de los valores del perfil hepático, la lactato-deshidrogenasa (765 Ul/L) y la ferritina (6.300 ng/ml); además de hipoalbuminemia, hipertrigliceridemia (375 mg/dl), leucopenia (3.620 células/μl) y trombocitopenia (53.000 células/μl). Se detectó infección por CMV, con una carga viral de 3.580 copias/ml. En el aspirado de médula ósea, se observaron macrófagos activados.

La paciente recibió inmunoglobulina humana inespecífica intravenosa y cumplió con el protocolo HLH-2004 completo. No se logró estudiar su componente genético. La paciente falleció por sepsis durante la aplicación del protocolo.

## Caso 4

Se trata de un niño de dos años, con síndrome febril y adenopatías cervicales, sin signos de alarma, por lo que se trató ambulatoriamente. Por la persistencia de los síntomas, fue nuevamente hospitalizado, con fiebre de 15 días de evolución y hepatoesplenomegalia.

Los resultados de los exámenes de laboratorio demostraron aumento de la ferritina (1.800 ng/ml), hipertrigliceridemia (267 mg/dl) y aumento de la lactato-deshidrogenasa (270 UI/L). Se realizó detección de carga viral de EBV con resultado positivo (500 copias/ml). El paciente presentó niveles normales de inmunoglobulinas séricas, y de linfocitos Ty B, y células NK.

Se identificaron macrófagos activados en el aspirado de médula ósea. Se practicó estudio genético de su exorna, en el cual se evidenció una variante homocigota probablemente patógena, en el gen *XIAP2* (p.Val80Lysfs*45).

En la junta médica, se decidió que el paciente era candidato a trasplante de progenitores hematopoyéticos, pero los padres rechazaron el procedimiento. Por lo anterior, se optó por iniciar tratamiento con inmunoglobulina humana inespecífica intravenosa y, por insistencia de la clínica, se inició el protocolo HLH-2004 completo y se obtuvo una adecuada evolución.

## Caso 5

Se trata de un niño de 22 meses de vida, con antecedentes de parto prematuro a las 36 semanas, oligohidramnios, retraso del desarrollo psicomotor, bajo peso para la edad gestacional, fisura palatina incompleta, malrotación intestinal corregida con portador de gastrostomía y artritis idiopática juvenil sistémica.

El paciente ingresó con un síndrome febril asociado con neutropenia (920 células/μl), anemia leve (hemoglobina de 11,8 g/dl), hepatomegalia y esplenomegalia, síndrome de dificultad respiratoria aguda, carditis e hiperferritinemia (46.069 ng/ml).

Se consideró el diagnóstico de síndrome hemofagocítico. El Servicio de Reumatologia inició tratamiento con metilprednisolona, inmunoglobulina humana inespecífica intravenosa y ciclosporina, al parecer con buena evolución clínica, por lo que fue dado de alta.

Posteriormente, el paciente fue hospitalizado en la Fundación Valle del Lili por presentar fiebre de un mes de evolución asociada con distensión abdominal. Se practicó una biopsia de médula ósea y no se detectaron macrófagos activados en la muestra. Los estudios inmunológicos evidenciaron valores normales de inmunoglobulinas y de células CD19, CD4 y NK, con niveles altos de CD8. Se practicó análisis solamente de su exorna en el cual se identificó una variante de significado clínico incierto en el gen *MAGT1* (c.183C/G), relacionada con deficiencia de magnesio, un error innato de la inmunidad que no explica el fenotipo del paciente. Finalmente, el paciente recibió manejo multidisciplinario con adecuada evolución.

## Caso 6

Se trata de una niña de cuatro años que fue remitida por presentar fiebre, astenia, adinamia y escalofríos. Tenía diagnósticos confirmados de dengue grave con derrame pleural y hepatitis, manejados en la unidad de cuidados intensivos. El Servicio de Infectología de la Fundación Valle del Lili solicitó pruebas de IgM e IgG para EBV, con resultado positivo para las dos.

Durante la hospitalización, presentó fiebre durante dos semanas, y dado que este no es un síntoma usual de la infección viral, se solicitaron estudios de extensión. Los estudios inmunológicos evidenciaron valores normales de inmunoglobulinas y de células T, B y NK, pero los exámenes de laboratorio revelaron aumento de los niveles de lactato-deshidrogenasa, hiperferritinemia (6.230 ng/ml), hipertrigliceridemia (367 mg/dl), "transaminitis" (sic.), hipoalbuminemia, aumento de la proteína C reactiva, anemia (hemoglobina, 8,5 g/dl) y trombocitopenia (121.000 plaquetas/μl). Estos resultados confirmaron el diagnóstico de síndrome hemofagocítico.

La paciente recibió manejo con dexametasona y transfusión de hemoderivados, con óptima respuesta terapéutica y una adecuada evolución clínica después de ser dado de alta.

## Caso 7

Se trata de una niña de nueve meses que presentaba tres días de fiebre, náuseas, emesis, astenia, adinamia y deposiciones líquidas, con hepatomegalia y un episodio convulsivo. Se evidenció infección por el virus del dengue y el SARS-CoV-2.

Durante su hospitalización, presentó fiebre persistente durante dos semanas, por lo cual se solicitaron estudios de extensión, encontrándose: anemia (hemoglobina, 9,8 g/dl), hiperferritinemia (3.373 ng/ml), fibrinógeno disminuido (95 mg/dl), hipertrigliceridemia (375 mg/dl), y aumento de las transaminasas, de la lactato-deshidrogenasa (1.317 Ul/L) y del dímero D. Tenía concentraciones normales de inmunoglobulinas y linfocitos T y B, pero había disminución de las células NK (50 células/μl).

La paciente recibió tratamiento con dexametasona y se obtuvo una buena respuesta terapéutica.

## Caso 8

Se trata de un niño de un año, con un cuadro clínico de tres meses de evolución de picos febriles intermitentes de predominio nocturno. Estuvo hospitalizado en otra institución, con diagnóstico de fiebre sin foco o sugestiva de bacteriemia oculta.

Fue valorado por infectología y se descartaron infecciones por el virus del dengue, HIV, sífilis, hepatitis B, SARS-CoV-2, leptospira y toxoplasma. Sin embargo, presentaba IgM positiva para citomegalovirus y para *Bartonella* spp.

Los exámenes de laboratorio de extensión evidenciaron leucocitosis (21.210 células/μl), anemia (hemoglobina, 9 g/dl), proteína C reactiva, aumento de la velocidad de sedimentación globular y del dímero D, hiperferritinemia (5.305 ng/ml), hipertrigliceridemia (382 mg/dl), valores normales de fibrinógeno y carga viral baja de CMV (368 copias/ml). La citometría de flujo de las células T demostró niveles de CD4 bajos para la edad, con niveles de CD8 y linfocitos B muy elevados. Los valores de las inmunoglobulinas séricas fueron normales.

En el examen físico, se detectó hepatomegalia. Por lo anterior, se sospechó un síndrome hemofagocítico, que fue confirmado mediante aspirado de médula ósea. EI paciente fue tratado con el protocolo HLH-2004 completo con adecuada evolución.

## Caso 9

Se trata de un niño de un año, que fue remitido por un cuadro clínico de síndrome febril de tres días de evolución, asociado con diarrea y emesis. En el examen físico, se encontró hepatoesplenomegalia, edema generalizado y deshidratación. Por los antecedentes epidemiológicos, se solicitó una prueba para anticuerpos IgG contra SARS-CoV-2, cuyo resultado fue positivo.

Los hallazgos de los exámenes de laboratorio evidenciaron: hiperferritinemia (3.623 ng/ml), hipoalbuminemia, hipertransaminasemia, lactato-deshidrogenasa elevada (1.260 Ul/L), leucocitosis (14.170 células/μl), anemia (hemoglobina, 8,9 g/dl) y trombocitopenia (59.000 células/μl).

Por lo anterior, se diagnosticó un síndrome inflamatorio multisistémico pediátrico (*Multisystem Inflammatory Syndrome in Children*, MIS-C) de tipo hemofagocítico, asociado con COVID-19.

Se inició tratamiento con pulsos de metilprednisolona e inmunoglobulina humana inespecífica intravenosa, No obstante, el paciente falleció.

## Caso 10

Se trata de un niño de un año, con un cuadro clínico de 13 días de fiebre, náuseas, emesis, inyección conjuntival, rinorrea, tos seca, edema periorbitario, astenia y adinamia, por lo cual consultó al servicio de urgencias.

En el examen físico, se encontró hepatoesplenomegalia, parálisis facial izquierda y signos sugestivos de otitis media izquierda. Se inició el tratamiento antibiótico. Los estudios de extensión indicaron una pansinusopatía con compromiso de la mastoides y del oído medio.

En los exámenes de laboratorio se encontró anemia (hemoglobina, 9 g/dl), fibrinógeno disminuido (119 mg/dl), hipertrigliceridemia (537 mg/dl) y "transaminitis" (sic.). Se consideró el diagnóstico de síndrome hemofagocítico. Se practicó una biopsia por aspirado de la médula ósea, en la que se detectaron macrófagos activados, con lo cual se confirmó el diagnóstico.

Los resultados de los estudios de inmunidad humoral y celular se encontraron en los rangos normales para la edad. El análisis de su exorna evidenció una variante homocigota del gen *STX11* (NM_003764.4): c.554dup (p.Trp186ValfsTer169), probablemente patógena para síndrome hemofagocítico familiar de tipo 4.

Se inició tratamiento con dexametasona e inmunoglobulina humana inespecífica intravenosa; se completó el protocolo HLH-2004 y se hizo trasplante de progenitores hematopoyéticos con adecuada evolución.

## Caso 11

Se trata de una niña de un año, con un cuadro clínico de cuatro días de evolución consistente en un síndrome febril asociado con erupción cutánea generalizada, náuseas, somnolencia e irritabilidad.

En el examen físico, se encontraron adenomegalias inguinales y petequias en el rostro y en las extremidades. Los resultados de los exámenes de laboratorio evidenciaron anemia (hemoglobina, 7,4 g/dl) y trombocitopenia (87.000 células/μl). La detección de IgM y NS1 del virus del dengue fue positiva.

A pesar del manejo de soporte, la paciente tuvo una evolución clínica complicada, por lo que fue trasladada a la unidad de cuidados intensivos donde se le practicaron los estudios de extensión.

La paciente presentó esplenomegalia, persistencia de la bicitopenia, hiperferritinemia (1.631 ng/ml), fibrinógeno disminuido (121 mg/dl), elevación de la lactato-deshidrogenasa (704 Ul/L), hipertrigliceridemia (388 mg/dl), disminución de las proteínas C*3* y C4, valores normales de IgG e IgA, pero con IgM elevada para la edad. Los linfocitos B y las células NK fueron normales, con disminución de CD4 y aumento de CD8.

Con estos hallazgos, se sospechó un síndrome hemofagocítico, posiblemente desencadenado por la infección con el virus del dengue y la coinfección confirmada con CMV (371.209 copias/ml). Se practicó una biopsia por aspirado de la médula ósea, en la que se encontraron macrófagos activados.

La paciente recibió tratamiento con dexametasona e inmunoglobulina humana inespecífica intravenosa. Sin embargo, por la persistencia de los síntomas, se inició el protocolo HLH-2004 completo. A pesar de todas las intervenciones, la paciente falleció.

## Caso 12

Se trata de un niño de tres años, con antecedentes de muerte de dos hermanos con diagnóstico de síndrome hemofagocítico secundario a infección por el virus de la varicela zóster y de Epstein-Barr, respectivamente.

El paciente ingresó por consulta externa para estudios de extensión por el antecedente familiar. El estudio genético demostró una variante hemicigota del gen *SH2D1A*: c.245_246insA (p.Asn82Lysfs*22), probablemente causante de un síndrome linfoproliferativo asociado con el cromosoma X. Los estudios de inmunidad celular y humoral demostraron rangos normales para la edad.

El paciente permanecía sin alteraciones clínicas ni de laboratorio en el momento del diagnóstico. No obstante, la junta médica decidió someterlo a un trasplante de progenitores hematopoyéticos y tuvo una buena evolución clínica.

## Discusión

El síndrome hemofagocítico se considera una enfermedad compleja que puede comprometer diferentes órganos. Su curso clínico es ambiguo, lo que hace que su identificación temprana sea un desafío que contribuye al subdiagnóstico y genera repercusiones en la atención clínica y en su caracterización global ^4^. Sin embargo, los niños menores de cinco años son un reto, no solo diagnóstico, sino etiológico. Por esta razón, el objetivo de este estudio fue describir y tratar de caracterizar los casos de síndrome hemofagocítico en menores de cinco años, tratados en un hospital de referencia del suroccidente de Colombia, entre enero del 2019 y diciembre del 2022.

En este periodo, se registraron 31 pacientes en la Fundación Valle del Lili de todos los grupos etarios, con diagnóstico de síndrome hemofagocítico. De ellos, 12 casos eran de menores de cinco años. De estos, 11 eran menores de 3 años, con una edad promedio de 1,4 años. Siete eran varones, lo que concuerda con la epidemiología global ^4^.

Las manifestaciones clínicas del síndrome hemofagocítico se caracterizan por fiebre, citopenias, organomegalias e hiperferritinemia ^5^. En el presente estudio, 11 pacientes presentaron fiebre, congruente con lo reportado en la literatura ^4^^,^^5^. El único paciente afebril recibió el trasplante antes de presentar sintomatologia, por sus antecedentes familiares. Además, se observó que la esplenomegalia estaba presente en 11 de los pacientes hospitalizados, lo cual coincide con los reportes internacionales ^5^ (cuadro 1).

**Cuadro 1:** Hallazgos clínicos y de laboratorio de la linfohistiocitosis hemofagocítica (N = 12)

Variable		Valor
Edad (años)		
	0	2
	1	6
	2	2
	3	1
	4	1
Sexo		
	Femenino	5
	Masculino	7
Enfermedad familiar		
	Desconocida	12
HLH2004: 5 de 8 criterios		
	Completos	11
Fiebre		
	Sí	11
	No	1
Esplenomegalia		
	Sí	11
	No	1
Estudio genético		7
	Negativo	2
	Positivo	5
Hemofagocitosis en médula ósea		
	Sí	5
	No	7
Leucocitos [mediana (IQR)]		8.200 (6.710- 16.943)
Neutrófilos [mediana (IQR)]		2.045(1.137,5-3032,5)
Hemoglobina [mediana (IQR)]		9,65 (8,98- 10,80)
Plaquetas [mediana (IQR)]		144.500 (80.500-306.750)
Fibrinógeno [mediana (IQR)]		209 (111 -292)
Triglicéridos [mediana (IQR)]		382 (371 -391)
Ferritina [mediana (IQR)]		3.623(1.716-6.265)
Lactato deshidrogenasa [mediana (IQR)]		789 (622 - 1.289)
Desencadenantes		
	Citomegalovirus	5
	Virus de Epstein-Barr	5
	Virus del dengue	2

IQR: rango intercuartílico

Había compromiso del sistema nervioso central en dos pacientes, en forma de convulsiones en el caso 7 y de compromiso facial en el caso 10. A este último se le practicó una tomografía computarizada de cráneo, en la cual no se apreciaron alteraciones significativas. Sin embargo, se le administró metotrexato intratecal como parte del tratamiento.

El compromiso neurológico fue menor en comparación con lo reportado en otras investigaciones. Jovanovic *et al*. reportaron que entre el 30 y el 50 % de los casos cursaban con estas manifestaciones ^6^. En una cohorte de 30 pacientes menores de cinco años, el mismo autor reportó que 17 presentaron manifestaciones neurológicas ^6^. Además, en nuestro estudio, se presentaron síntomas gastrointestinales solo en 4 de los niños participantes, una proporción menor que la documentada ^3^.

El síndrome hemofagocítico presenta un amplio espectro de agentes causales y factores desencadenantes, entre los cuales se destacan los procesos infecciosos con diversas presentaciones clínicas e importantes tasas de morbimortalidad, las cuales varían según la predisposición inmunológica del huésped.

En el presente estudio, se observó una frecuencia significativa de infección viral como factor desencadenante (10 casos). El virus de Epstein- Barr y el citomegalovirus fueron los principales agentes causales, presentes en cinco pacientes cada uno. También, se registraron infecciones por el virus del dengue y SARS-CoV-2 (n = 2 cada uno). Estos hallazgos concuerdan con los de otros reportes previos ^(3, 7-9)^ que mencionan al CMV y al EBV como desencadenantes del síndrome hemofagocítico. Estos virus, sumados a la presentación clínica de la enfermedad y a la edad, sugieren el subtipo primario del síndrome hemofagocítico.

La bicitopenia o pancitopenia es extremadamente común en niños que presentan síndrome hemofagocítico ^3^^,^^6^. En el presente trabajo, la bicitopenia se presentó en seis pacientes, mientras que la pancitopenia lo hizo en dos. Las inmunoglobulinas séricas se evaluaron en once pacientes y solo uno presentó hipogammaglobulinemia, únicamente a expensas de la IgG, mientras que los demás presentaron niveles normales de IgAe IgM. Es importante destacar que los pacientes menores de cinco años que cursan con hipogammaglobulinemia y síndrome hemofagocítico, podrían tener el subtipo primario 4 de la enfermedad. En este reporte, el caso 1 presentó infección por EBV, pero el estudio de su exorna resultó sin variantes patógenas, por lo cual se descartaron alteraciones genéticas primarias.

En el presente estudio, se evaluó la ferritina sérica en todos los pacientes con diagnóstico de síndrome hemofagocítico, a excepción del caso 12, que no presentó alteraciones clínicas ni de laboratorio. Esta práctica está respaldada por el protocolo institucional, cuyas directrices indican la medición de este parámetro en todos los pacientes que presenten fiebre sin foco claro o prolongada (más de cinco días), evolución tórpida o sepsis resistente.

Todos los pacientes evaluados, 11 en total, presentaron hiperferritinemia, con una mediana de 3.623 ng/ml y un rango intercuartílico de 632 a 46.069 ng/ml. Este hallazgo es similar a los resultados de una serie de casos de 18 pacientes en España, en la cual se reportó que todos los pacientes pediátricos fallecidos presentaban hiperferritinemia ^8^. En otra cohorte de 369 pacientes con síndrome hemofagocítico, el 88 % presentaba esta alteración ^5^. A pesar de que todos los pacientes de este reporte presentaron hiperferritinemia, llama la atención que aquellos que fallecieron no tenían niveles de ferritina superiores a 20.000 ng/ml, valores que han sido previamente asociados con mayor mortalidad.

Se practicó biopsia por aspirado de médula ósea en todos los pacientes, para determinar la presencia de macrófagos activados y esta población celular se detectó en 5 casos. Esto se correlaciona con lo informado por otros autores quienes reportan macrófagos activados en el 40 al 80 % de los casos, según el momento de la toma de muestra ^10^^-^^13^. Sin embargo, este hallazgo puede ser secundario en el curso del síndrome.

Las pruebas genéticas se llevan a cabo en situaciones específicas, especialmente en pacientes menores de cinco años, en quienes la probabilidad de síndrome hemofagocítico primario es mayor. Estas pruebas constituyen un pilar esencial en la investigación médica de estas enfermedades. A todos los pacientes se les solicitó estudio genético, sin embargo, solo se les practicó a 8: en 2 de ellos, no se evidenciaron variantes patógenas; asimismo, se encontraron 4 casos con variantes asociadas con el síndrome hemofagocítico primario y 1 (caso 5) con una variante relacionada con otro tipo de error innato de la inmunidad (además de síndrome hemofagocítico) que no se correlaciona con su fenotipo (cuadro 2).

**Cuadro 2:** Resultados de los estudios genéticos

Caso	Estudio genético	Variantes encontradas	Desenlace
1	Exorna NSG	Ninguna	Vivo
2	Exorna NSG	Mutación del gen *PRF1*, 10q22.1 (NM_001083116.3) C.160OT (p.Arg54Cys)	Vivo
		Variante heterocigota, probablemente patógena	
		Mutación del gen *PRF1*, 10q22.1 (NM_001083116.3) c.350_356delTGGCCCGinsATGC (p.Val117_	
		Argil 9delinsAspAla)	
		Variante heterocigota, probablemente patógena	
4	Exorna NSG	Mutación del gen XIAP	Vivo
		Variante homocigota, probablemente patógena (p.Val80Lysfs*45)	
		Pérdida de función del gen XIAP	
5	Exorna NSG	Mutación de significado incierto del gen MAGT1, C.183C/G asociada con alteración congénita de la glicosilación de tipo ICC, inmunodeficiencia ligada al cromosoma X, con deficiencia de magnesio, infección por virus de Epstein-Barr y neoplasia	Vivo
10	Exorna NSG	Mutación del gen *STX11* (NM 003764.4) c.554dup (p.TrpI 86ValfsTer169)	Vivo
		Variante homocigota, probablemente patógena, asociada con linfohistiocitosis hemofagocítica familiar de tipo 4	
12	Exorna NSG	Mutación del gen *SH2D1A* c.245_246insA (p.Asn82Lysfs*22) Variante hemicigota, probablemente patógena	Vivo

NGS: *Next Generation Sequencing*

Esto contrasta con lo encontrado por Chinn *et al*., quienes practicaron estudios genéticos a 101 de 122 pacientes pediátricos (87,7 %). De estos, 35 no presentaron variantes bialélicas en los genes relacionados con el desarrollo de síndrome hemofagocítico familiar; se identificaron 20 pacientes con variantes genéticas de significado clínico incierto, 19 con alteraciones en genes asociados con síndrome hemofagocítico familiar, 11 con variantes asociadas a errores innatos de la inmunidad, 8 con anomalías en genes relacionados con la activación o proliferación inmunitaria desregulada y 3 con variantes vinculadas a las últimas dos enfermedades mencionadas.

Esta disparidad de los estudios genéticos en la cohorte del presente trabajo comparada con la de otros estudios, puede deberse al sistema de salud colombiano, el cual proporciona poco acceso a este tipo de estudios y está influenciado por la escasa sospecha clínica de esta entidad ^14^.

Con estos resultados, es difícil concluir si todos o la mayoría de los casos corresponden a síndrome hemofagocítico primario, pero sí es claro que, a todos los pacientes menores de cinco años con este síndrome, se les debe solicitar estudios genéticos desde el momento del diagnóstico, lo cual permitirá un acceso oportuno a un trasplante de progenitores hematopoyéticos.

Una vez establecido el diagnóstico del síndrome hemofagocítico, el objetivo fundamental del tratamiento consiste en suprimir y controlar la hiperinflamación e hipercitocinemia, además de eliminar las células activadas e infectadas.

La implementación de esquemas terapéuticos basados en etopósidos ha aumentado sustancialmente la supervivencia ^9^; por esta razón, la evidencia sugiere implementar el protocolo HLH-2004 independientemente de si el desencadenante del síndrome hemofagocítico es primario o secundario ^6^. Algunos evidencian mejores resultados en presencia de infecciones por EBV al orientar el tratamiento según el protocolo HLH-2004 ^6^. Cinco pacientes recibieron el tratamiento completo según el protocolo HLH-2004, de los cuales 2 fallecieron. El resto de los pacientes recibió un tratamiento heterogéneo que varió entre esteroides, ciclosporina e inmunoglobulina humana inespecífica intravenosa (cuadro 3).

**Cuadro 3.** Tratamiento

Caso	Tratamiento	Trasplante de medula ósea	Desenlace
1	Protocolo HLH-2004 e IGIV	No	Vivo
2	Protocolo HLH-2004 e IGIV	Sí	Vivo
3	Protocolo HLH-2004 e IGIV	No	Fallecido
4	IGIV y dexametasona	No	Vivo
5	IGIV, dexametasona y ciclosporina	No	Vivo
6	Dexametasona	No	Vivo
7	Dexametasona	No	Vivo
8	Protocolo HLH-2004 e IGIV	No	Vivo
9	IGIV y dexametasona	No	Fallecido
10	Protocolo HLH-2004 e IGIV	Sí	Vivo
11	IGIV y dexametasona	No	Fallecido
12	Sin tratamiento	Sí	Vivo

IGIV: inmunoglobulina humana inespecífica intravenosa

Hasta la fecha, el trasplante de progenitores hematopoyéticos se considera como el único tratamiento curativo conocido para los casos de síndrome hemofagocítico primario o secundario resistente al tratamiento ^7^. En este contexto, se plantea la relevancia de considerar una intervención temprana mediante el trasplante de progenitores hematopoyéticos en pacientes asintomáticos que portan variantes patógenas o presentan antecedentes familiares de síndrome hemofagocítico ^13^. El caso 12 de esta serie ejemplifica esta perspectiva asintomática y sin alteraciones de laboratorio, pero con una historia familiar de dos fallecimientos previos, secundarios a síndrome hemofagocítico primario. El estudio genético reveló la presencia de la variante patógena, c.245_246insA (p.Asn82Lysfs*22), identificada en el gen *SH2D1A*, asociado con síndrome hemofagocítico y errores innatos de la inmunidad.

Este enfoque preventivo puede contribuir significativamente a mejorar los resultados del tratamiento a largo plazo y la calidad de vida de los pacientes afectados ^13^. Tres fueron sometidos a trasplante de progenitores hematopoyéticos y todos sobrevivieron.

Al considerar la presentación clínica inespecífica y la escasa prevalencia del síndrome hemofagocítico, pero la gran probabilidad de efectos adversos para los pacientes, la inclusión temprana del diagnóstico diferencial al síndrome hemofagocítico puede disminuir el riesgo de hacer diagnósticos tardíos y mejorar los resultados clínicos de los pacientes, sobre todo en los menores de cinco años. Es importante resaltar que, aunque no todos los pacientes tuvieron estudio genético, sí es necesario realizarlo de forma oportuna en los menores de cinco años para lograr un diagnóstico temprano y preciso, y, dado el caso, decidir oportunamente sobre un posible trasplante de progenitores hematopoyéticos.

No es claro si el inicio del protocolo HLH-2004 en todos pacientes de este reporte influyó en su evolución, pero, en definitiva, las decisiones sobre tratamientos e intervenciones, más en el contexto colombiano, deben ser rápidas y claras, particularmente en esta población de menores de cinco años, ya que puede que no todos respondan satisfactoriamente al tratamiento inicial con dexametasona o inmunoglobulina humana inespecífica intravenosa.

En conclusión, en este estudio se resalta la complejidad del síndrome hemofagocítico en los niños menores de cinco años, en quienes la baja prevalencia de la enfermedad y la presentación clínica inespecífica contribuyen a su subdiagnóstico. Los hallazgos del presente estudio subrayan la importancia de considerar una amplia gama de factores desencadenantes -como las infecciones virales-y la evaluación genética para un diagnóstico preciso.

Aunque el protocolo HLH-2004 y el trasplante de progenitores hematopoyéticos son intervenciones fundamentales, su efectividad depende de la edad del paciente y de la gravedad de la enfermedad. Se destaca la necesidad de un enfoque multidisciplinario y la consideración oportuna de intervenciones como el trasplante de progenitores hematopoyéticos, para mejorar la morbimortalidad en estos pacientes.
